# Heterologous synthesis of a simplified nitrogenase analog in *Escherichia coli*

**DOI:** 10.1126/sciadv.adw6785

**Published:** 2025-05-02

**Authors:** Yiling A. Liu, Chi Chung Lee, Kamil Górecki, Martin T. Stiebritz, Calder Duffin, Joseph B. Solomon, Markus W. Ribbe, Yilin Hu

**Affiliations:** ^1^ Department of Molecular Biology and Biochemistry, University of California, Irvine, CA 92697-3900, USA.; ^2^ Department of Biology, Friedrich-Alexander University Erlangen-Nuremberg, D-91052 Erlangen, Germany.; ^3^ Department of Chemistry, University of California, Irvine, CA 92697-2025, USA.

## Abstract

The heterologous synthesis of a nitrogen-fixing system in a non-diazotrophic organism is a long-sought-after goal because of the crucial importance of nitrogenase for agronomy, energy, and the environment. Here, we report the heterologous synthesis of a two-component nitrogenase analog from *Azotobacter vinelandii*, which consists of the reductase component (NifH) and the cofactor maturase (NifEN), in *Escherichia coli*. Metal, electron paramagnetic resonance, and activity analyses verify the cluster composition and functional competence of the heterologously expressed NifH and NifEN. Nuclear magnetic resonance, nanoscale secondary ion mass spectrometry, and growth experiments further illustrate the ability of the NifH/NifEN system to reduce N_2_ and incorporate the reduced N into the cellular mass. These results establish NifEN/NifH as a simplified nitrogenase analog that could be optimized and engineered to facilitate transgenic expression and biotechnological adaptations of this important metalloenzyme.

## Teaser

Heterologous synthesis of a nitrogenase analog (NifH/NifEN) in *E. coli* enables N_2_ reduction and incorporation of N into biomass.

## INTRODUCTION

Nitrogenase catalyzes the ambient conversion of N_2_ to NH_3_, a key step in the global nitrogen cycle that is crucial for all life forms on Earth (*1*–*4*).The “conventional” Mo-nitrogenase consists of (i) a reductase component (termed Fe protein or NifH), which is a homodimer containing a [Fe_4_S_4_] cluster at the subunit interface and a magnesium adeonsine triphosphate (MgATP) binding site within each subunit; and (ii) a catalytic component (termed MoFe protein or NifDK), which is an α_2_β_2_-heterotetramer containing a P-cluster ([Fe_8_S_7_]) at each α/β-subunit interface and an M-cluster {also known as the cofactor; [(*R*-homocitrate)MoFe_7_S_9_C]} within each α subunit (fig. S1A, left) (*5*–*9*). Catalysis by the Mo-nitrogenase involves repeated association and dissociation between NifH and NifDK, which permits adenosine 5′-triphosphate (ATP)–dependent, interprotein electron transfer in the direction of [Fe_4_S_4_] cluster (NifH) → P-cluster (NifDK) → M-cluster (NifDK), followed by substrate reduction at the M-cluster upon accumulation of a sufficient number of electrons (fig. S1A, left) (*2*–*6*). Such a two-component system is highly versatile and capable of reducing a variety of substrates, including CO, C_2_H_2_, N_3_^−^, and H^+^ (fig. S1A, left) (*1*, *10*–*12*). Notably, the reduction of CO to hydrocarbons by nitrogenase parallels the production of carbon fuels by the industrial Fischer-Tropsch process (*13*, *14*), much like the parallelism between the reduction of N_2_ to NH_3_ by nitrogenase and the production of ammonia by the industrial Haber-Bosch process (*15*, *16*). Yet, contrary to their respective industrial parallels, both enzymatic reactions occur at ambient conditions and use protons/electrons as the reducing equivalents, making nitrogenase an attractive target for developing energy- and environment-friendly strategies for the production of valuable chemical commodities in the future.

Underscoring the catalytic prowess of the Mo-nitrogenase is its active-site M-cluster, arguably the most complex metallocofactor found in nature that has, thus far, evaded successful chemical synthesis. Biosynthesis of this complex metallocofactor (fig. S1B) starts with mobilization of Fe and S by NifS (a cysteine desulfurase) and NifU (an FeS assembly scaffold) for the formation of small [Fe_4_S_4_] units (*11*, *17*, *18*). This event is followed by transfer of a pair of [Fe_4_S_4_] clusters to NifB [a radical *S*-adenosyl-l-methionine (SAM) enzyme], which transforms the [Fe_4_S_4_] cluster pair (termed K-cluster) into an [Fe_8_S_9_C] cluster (termed L-cluster) concomitant with the incorporation of a SAM-derived interstitial carbide and a sulfite-derived “ninth” sulfide (*9*, *19*–*21*). Subsequently, the L-cluster is delivered to NifEN (a cofactor maturase) and converted to a fully assembled M-cluster via NifH-mediated insertion of Mo and homocitrate (*22*–*24*) before transfer of the M-cluster to its destined location in apo-NifDK, which marks the completion of the biosynthesis of the M-cluster concomitant with the formation of a holo-NifDK (*11*, *25*).

### Homology between NifEN and NifDK

There is a notable resemblance between NifEN (a key player in biosynthesis) and NifDK (an essential component in catalysis) in terms of their sequences, tertiary structures, and associated metallocenters. Crystallographic analysis of an L-cluster–bound form of *Azotobacter vinelandii* NifEN (designated *Av*NifEN^L^) has revealed an α_2_β_2_-heterotetramic conformation of NifEN that is homologous to that of its *Av*NifDK counterpart (fig. S1A, right) (*26*), as well as the presence of a pair of analogous clusters in *Av*NifEN^L^ to the P- and M-clusters in *Av*NifDK at similar locations: (i) an O-cluster ([Fe_4_S_4_]) at the α/β-subunit interface, which can be seen as one half of the P-cluster ([Fe_8_S_7_]), and (ii) an L-cluster ([Fe_8_S_9_C]) within the α subunit, which is structurally nearly indistinguishable from the M-cluster but has an Fe atom instead of Mo/homocitrate at one end of the cluster (fig. S1B). It is conceivable, therefore, that *Av*NifEN^L^ could be paired with *Av*NifH into a two-component system analogous to that comprising *Av*NifDK and *Av*NifH, which permits an ATP-dependent electron flow in the direction of [Fe_4_S_4_] (*Av*NifH) → O-cluster (*Av*NifEN^L^) → L-cluster (*Av*NifEN^L^) to enable substrate reduction at the active-site L-cluster (fig. S1A, right).

Consistent with this proposal, we demonstrated previously the ability of *Av*NifEN^L^ to mimic its *Av*NifDK counterpart and reduce C_2_H_2_ and N_3_^−^ (fig. S1A, right), the “easier” alternative substrates, in an in vitro assay containing *Av*NifH, ATP, and dithionite (*27*, *28*). In a recent work, we illustrated the ability of *Av*NifEN^L^ to enable the in vitro reduction of N_2_, the more “difficult” physiological substrate, to NH_3_ at a turnover number of 0.8 in an ATP-dependent assay containing *Av*NifH and dithionite (*29*). These observations are exciting, as they point to a distinct possibility to simplify the biosynthesis of nitrogenase by replacing the catalytic component NifDK with the catalytically competent NifEN, thereby omitting the need to mature the L-cluster into an M-cluster on NifEN and subsequently insert the M-cluster into an apo-NifDK scaffold (fig. S1B). Such a simplification in the number of biosynthetic components and events would greatly facilitate the heterologous expression of nitrogenase in a foreign host, an actively pursued topic given its potential of enabling future efforts to express nitrogenase in higher organisms or developing nitrogenase-based biotechnological applications.

## RESULTS

### Synthesis of a simplified nitrogenase analog comprising NifH/NifEN in *Escherichia coli*

Along the line of introducing nitrogenase into a non-diazotrophic organism, we used a bottom-up, metallo-centric strategy in our previous studies to individually tackle the metallocluster composition and biosynthetic competence of the key components of nitrogenase assembly (*30*–*32*) before combining these components for the heterologous expression of an active Mo-nitrogenase (i.e., NifH/NifDK) in *E. coli* (*33*). Building on the success of these studies, we co-expressed the *nifH*,*M*,*E*,*N* genes from *A. vinelandii* and the *nifS3*,*U3*,*B* genes from *Methanosarcina acetivorans* in *E. coli* strain MY21, a BL21(DE3)-derived Δ*iscR* strain, which should allow for the heterologous synthesis of a two-component nitrogenase analog (i.e., NifH/NifEN^L^) via the expression of (i) a [Fe_4_S_4_] cluster–bound NifH (requiring *nifH*,*M*), (ii) an L-cluster–bound NifB (requiring *nifS3*,*U3*,*B*), and (iii) an O-cluster–replete but L-cluster–depleted NifEN^apo^ (requiring *nifE*,*N*), followed by an in vivo transfer of the L-cluster from NifB to NifEN to yield an O- and L-cluster–replete NifEN^L^ as a crucial event of cofactor assembly (see fig. S1B).

**Fig. 1.**
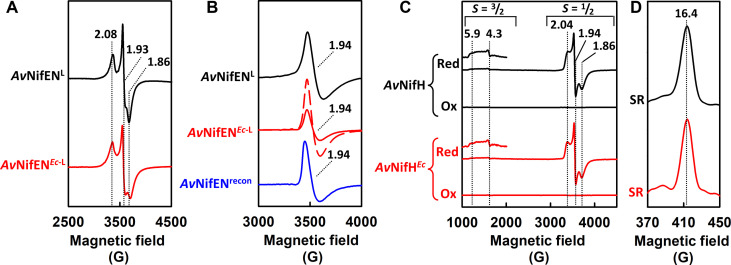
Spectroscopic characterization of *Av*NifEN^*Ec*-L^ and *Av*NifH*^Ec^*. (**A**) Perpendicular-mode electron paramagnetic resonance (EPR) spectra of dithionite-reduced *Av*NifEN^L^ (black) and *Av*NifEN^*Ec*-L^ (red). The *g* values of the composite *S* = ^1^/_2_ signal that originates from the O- and L-clusters are indicated. (**B**) Perpendicular-mode EPR spectra of the IDS-oxidized *Av*NifEN^L^ (black solid), *Av*NifEN^*Ec*-L^ (red solid), and *Av*NifEN^recon^ (blue solid), showing the L-cluster–specific signal at *g* = 1.94. Also shown is the EPR spectrum of *Av*NifEN^*Ec*-L^ normalized on the basis of the L-cluster content (red dashed). The EPR signals were identified through benchmarking against well-established reference signals (*31*, *34*). (**C**) Perpendicular- and (**D**) parallel-mode EPR spectra of oxidized (Ox), reduced (Red), and super-reduced (SR) *Av*NifH (black) and *Av*NifH*^Ec^* (red). The three oxidation states of *Av*NifH or *Av*NifH*^Ec^* were generated upon treatment of the protein with IDS (Ox), dithionite (Red), and Eu^II^-EGTA (SR), respectively. The *S* = ^3^/_2_ signals observed in the spectra of both *Av*NifH and *Av*NifH*^Ec^* in the reduced state are also shown at fivefold enhanced intensities above the respective spectra. The EPR signals were identified through benchmarking against well-established reference signals (*1*, *10*).

When co-expressed anaerobically with a non-tagged form of NifH, a His-tagged form of *A. vinelandii* NifEN (designated *Av*NifEN^*Ec*-L^) could be isolated anaerobically by immobilized metal affinity chromatography (IMAC) from *E. coli* expression strain YM578EE as a soluble, brown protein at a yield of ~500 mg per 100 g of wet cells. The as-isolated *Av*NifEN^*Ec*-L^ is an α_2_β_2_-tetramer comprising α and β subunits of ~50 and ~49 kDa, respectively (fig. S2A). Like its native *Av*NifEN^L^ counterpart (Fig. 1A, black), the heterologously expressed *Av*NifEN^*Ec*-L^ (Fig. 1A, red) exhibited a composite *S* = ^1^/_2_ (*S*, spin quantum number) perpendicular-mode electron paramagnetic resonance (EPR) signal in the dithionite-reduced state, which originated from both the O- and L-clusters (*31*, *34*). In the indigo disulfonate (IDS)–oxidized state, *Av*NifEN^*Ec*-L^ (Fig. 1B, solid red) displayed the same *g* = 1.94 (*g*, *g*-factor) perpendicular-mode EPR signal as its native *Av*NifEN^L^ counterpart (Fig. 1B, black), which originated specifically from the L-cluster (*31*, *34*). The presence of an L-cluster in the heterologously expressed *Av*NifEN^*Ec*-L^ was further verified by extracting the L-clusters into an organic solvent, *N*-methylformamide (NMF), and subsequently reconstituting the O-cluster–replete yet L-cluster–depleted apo *Av*NifEN, as this procedure resulted in a reconstituted *Av*NifEN species (designated *Av*NifEN^recon^) that displayed the same L-cluster specific, *g* = 1.94 EPR signal (Fig. 1B, blue) as that displayed by *Av*NifEN^L^ (Fig. 1B, black) or *Av*NifEN^*Ec*-L^ (Fig. 1B, solid red). A comparison of the intensities of the *g* = 1.94 features indicated an L-cluster occupancy of 36% in *Av*NifEN^*Ec*-L^ relative to that in the native *Av*NifEN^L^ (Fig. 1B), consistent with the Fe content-based calculation of the L-cluster occupancy (42.9%) alongside a full complement of the O-clusters in *Av*NifEN^*Ec*-L^ (fig. S2B), as well as the relative activities of *Av*NifEN^*Ec*-L^ to its native counterpart in maturation (40.1%; fig. S2C) and C_2_H_2_ reduction (39.3%; fig. S2C). Such an L-cluster content of *Av*NifDK^*Ec*-L^ illustrates a good cross-species interaction between *Ma*NifB and *Av*NifEN, which permitted an efficient in vivo transfer of the L-cluster from the former to the latter as a key step in the heterologous expression of a holo NifEN species in *E. coli*.

Having established the successful expression of *Av*NifEN^L^ in *E. coli*, we then verified the co-expression of *Av*NifH in this foreign host by constructing an *E. coli* expression strain YM586EE, which was identical to YM578EE except that both NifH and NifEN contained a polyhistidine tag. This strategy allowed for the anaerobic co-expression and co-purification of His-tagged NifH (designated *Av*NifH*^Ec^*) and His-tagged NifEN (*Av*NifEN^*Ec*-L^) by IMAC, followed by further purification of *Av*NifH*^Ec^* by size exclusion chromatography (SEC), as a soluble, brown protein at a yield of ~65 mg per 100 g of wet cells. The as-isolated *Av*NifH^*Ec*-L^ is a γ_2_-homodimer comprising identical subunits of ~30 kDa (fig. S2D). Like the native *Av*NifH (Fig. 1, C and D, black), the heterologously expressed *Av*NifH*^Ec^* (Fig. 1, C and D, red) was capable of adopting three oxidations states: the reduced, [Fe_4_S_4_]^+^ state that was characterized by mixed *S* = ^3^/_2_:*S* = ^1^/_2_ EPR signals (Fig. 1C, “Red”); the oxidized, [Fe_4_S_4_]^2+^ state that was EPR silent (Fig. 1C, “Ox”); and the super-reduced, [Fe_4_S_4_]^0^ state that displayed a unique, *g* = 16.4 EPR feature (Fig. 1D, “SR”) (*32*). Moreover, consistent with a cluster content that was 78% of that of the native *Av*NifH (fig. S2E), *Av*NifH*^Ec^* showed 79.5% activity in C_2_H_2_ reduction relative to that of its native counterpart (fig. S2F).

### In vitro N_2_ reduction by the nitrogenase analog comprising NifH/NifEN

**Fig. 2.**
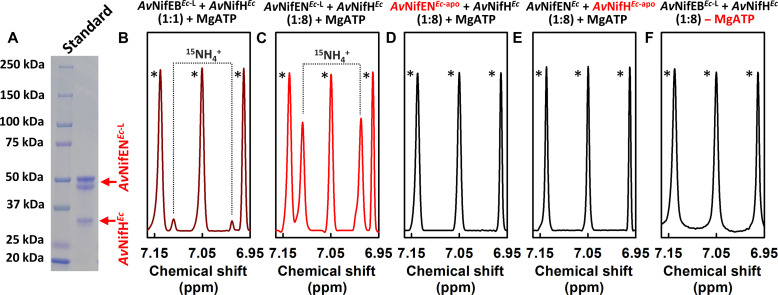
ATP-dependent formation of ammonia by *Av*NifH^*Ec*-L^. (**A**) SDS–polyacrylamide gel electrophoresis of *Av*NifH*^Ec^* and *Av*NifEN^*Ec*-L^ co-purified from *E. coli* strain YM586EE. (**B** and **C**) Frequency-selective pulse ^1^H NMR spectra of ^15^NH_4_^+^ generated upon ATP-dependent reduction of ^15^N_2_ by (B) *Av*NifH*^Ec^* and *Av*NifEN^*Ec*-L^ co-purified at a protein molar ratio of ~1:1 and (C) *Av*NifH*^Ec^* and *Av*NifEN^*Ec*-L^ individually isolated and mixed at a protein molar ratio of 8:1. The turnover numbers are 0.2 and 3, respectively, for experiments depicted in (B) and (C). (**D** to **F**) Control assays with (D) *Av*NifEN^*Ec*-L^ replaced by *Av*NifEN^*Ec*-apo^, (E) *Av*NifH*^Ec^* replaced by *Av*NifH^*Ec*-apo^, and (F) MgATP omitted. Note that the triplet signals in the NMR spectra (labeled with *) represent the ^14^NH_4_^+^ background generated upon protein degradation.

Excitingly, an IMAC fraction from the expression strain YM586EE containing co-purified His-tagged *Av*NifH^*Ec*-L^ and His-tagged *Av*NifH^*E*c^ at a molar ratio of ~1:1 (Fig. 2A) was capable of ATP-dependent reduction of ^15^N_2_ to ^15^NH_3_ in the presence of dithionite, as reflected by the ^15^NH_4_^+^-specific doublet at 6.99 and 7.11 parts per million (ppm) in the frequency-selective pulse ^1^H nuclear magnetic resonance (NMR) spectrum (Fig. 2B) (*35*). The same reactivity could be achieved by combining *Av*NifEN^*Ec*-L^ that was individually isolated from strain YM578EE with *Av*NifH*^Ec^*, ATP, and dithionite (Fig. 2C); only in this case, a much stronger intensity of the ^15^NH_4_^+^-specific doublet could be achieved when *Av*NifH*^Ec^* was supplied at an 8:1 protein molar excess to *Av*NifEN^*Ec*-L^ (with 36% L-cluster occupancy), a condition mimicking the molar excess of NifH to NifDK in a standard, in vitro activity assay of nitrogenase. Of note, no ^15^NH_4_^+^ could be detected (i) when the heterologously expressed, O-cluster–containing yet L-cluster–deficient apo *Av*NifEN (designated *Av*NifEN^*Ec*-apo^) was used in place of *Av*NifEN^*Ec*-L^ (Fig. 2D); (ii) when a heterologously expressed, [Fe_4_S_4_]-deficient apo *Av*NifH (designated *Av*NifH^*Ec*-apo^) was used in place of *Av*NifH*^Ec^* (Fig. 2E); or (iii) when ATP was omitted from the reaction (Fig. 2F). These results align well with the observations derived from the native *Av*NifH/*Av*NifEN^L^ proteins (*29*) and firmly establish the L-cluster in *Av*NifEN^*Ec*-L^ as the origin of the N_2_-reducing activity while pointing to *Av*NifH as an ATP-dependent electron donor for *Av*NifEN in this reaction. Moreover, the ability of the co-purified *Av*NifH*^Ec^*/*Av*NifEN^*Ec*-L^ proteins to perform in vitro N_2_ reduction in the presence of dithionite (see Fig. 2B) points to a distinct possibility of the *E. coli* strain co-expressing these proteins to use *Av*NifH*^Ec^*/*Av*NifEN^*Ec*-L^ as a two-component nitrogenase analog for the in vivo N_2_ reduction in the presence of an appropriate electron donor.

**Fig. 3.**
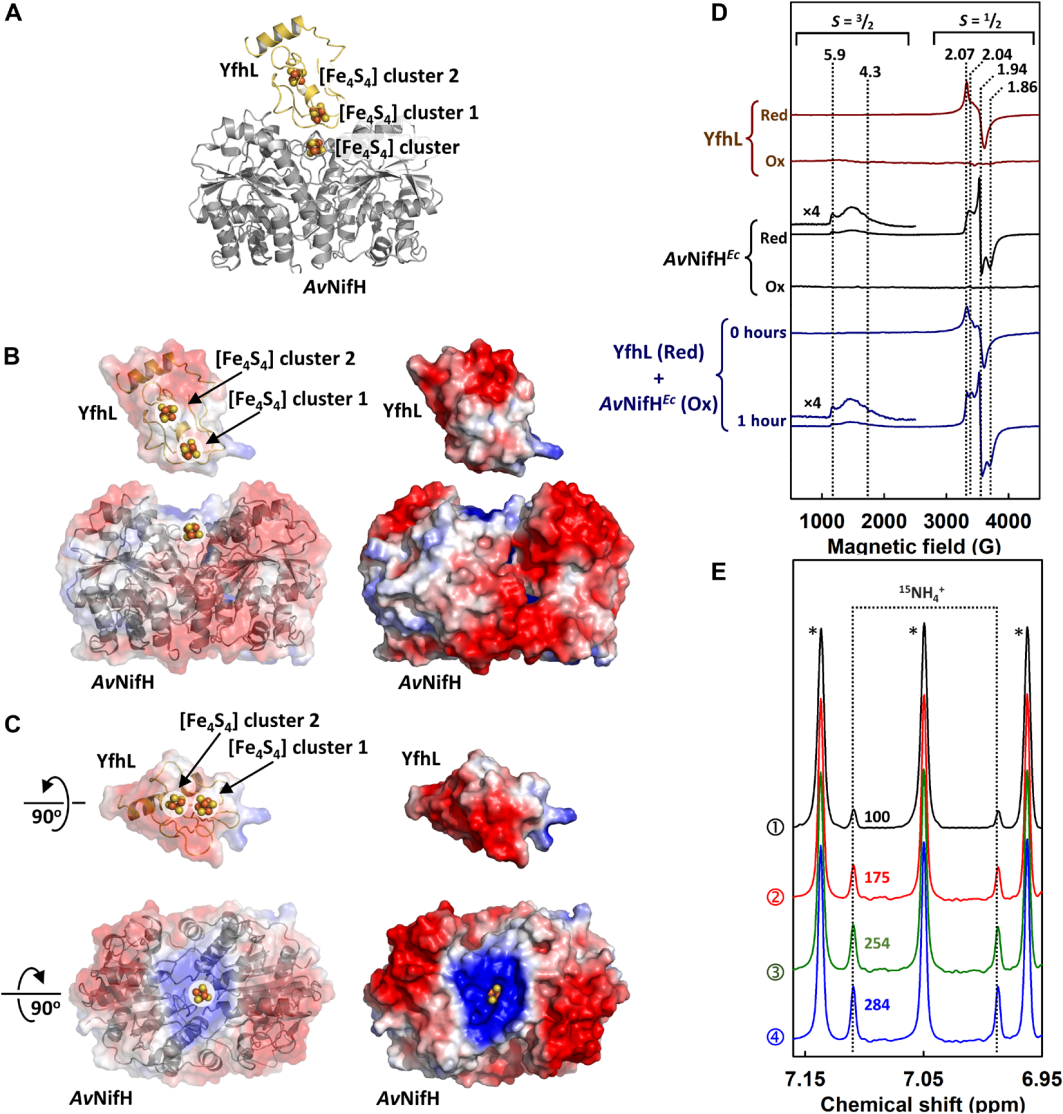
Interaction between *Av*NifH and YfhL. (**A** to **C**) Cartoon (A) and surface [(B) and (C)] presentations of an energy-optimized model of the *Av*NifH/YfhL complex. The model shows a strong docking energy of −13.3 kcal/mol with a minimum distance of 7.2 Å between the [Fe_4_S_4_] cluster of *Av*NifH and [Fe_4_S_4_] cluster 1 of YfhL. *Av*NifH [Protein Data Bank (PDB) entry 2NIP] and YfhL (PDB entry 2ZVS) are colored gray and yellow, respectively (A), and the negative and positive surface charges of these proteins are colored red and blue, respectively [(B) and (C)]. [(B) and (C)] The surface is rendered (left) transparent to indicate the position of the [Fe_4_S_4_] clusters and (right) solid to emphasize on the surface charge. The [Fe_4_S_4_] clusters are shown as balls and sticks, with Fe and S colored orange and yellow, respectively. (**D**) EPR analysis of electron transfer between *Av*NifH*^Ec^* and YfhL. Shown are the perpendicular-mode EPR spectra of dithionite-reduced (Red) and IDS-oxidized (Ox) YfhL (brown) and *Av*NifH*^Ec^* (black). Upon mixing of the reduced YfhL and the oxidized *Av*NifH*^Ec^*, only the *S* = ^1^/_2_ EPR signal of the reduced [Fe_4_S_4_]^+^ cluster of YfhL was observed (dark blue, 0 hours). Incubation of this mixture for 1 hour resulted in a transfer of electrons from the reduced YfhL to the oxidized *Av*NifH*^Ec^*, as indicated by the appearance of a mixed *S* = ^3^/_2_:*S* = ^1^/_2_ spin state of the reduced [Fe_4_S_4_]^+^ cluster of *Av*NifH*^Ec^* (dark blue, 1 hour). The EPR signals were identified through benchmarking against well-established reference signals (*1*, *10*, *57*). (**E**) Frequency-selective pulse ^1^NMR spectra of ^15^NH_4_^+^ generated upon ATP-dependent reduction of ^15^N_2_ by *Av*NifH*^Ec^* and *Av*NifEN^*Ec*-L^ in the presence of 0.3 mM dithionite (①) or an equimolar (②), twofold molar excess (③), or threefold molar excess (④) of reduced YfhL. Note that the triplet signals in the NMR spectra (labeled with *) represent the ^14^NH_4_^+^ background generated upon protein degradation.

Because no exogenous ferredoxin or flavodoxin was introduced into the *E. coli* expression strain (YM578EE) alongside *Av*NifH*^Ec^* and *Av*NifEN^*Ec*-L^, we examined the endogenous ferredoxins and flavodoxins of *E. coli* and identified YfhL as the most promising candidate to serve as an in vivo electron donor for *Av*NifH. Sharing a high degree of sequence homology (47%) with *Av*FdxN, a ferredoxin involved in nitrogen fixation, the two [Fe_4_S_4_] clusters of YfhL are poised at redox potentials (*E*^0^ = −675 mV for [Fe_4_S_4_] cluster 1 and −418 mV for [Fe_4_S_4_] cluster 2; *E*^0^, standard reduction potential) (*36*) that are well suited for enabling electron transfer to *Av*NifH (*E*^0^ = −405 mV) (*37*). Using a previously described approach (*38*), a combined docking/energy evaluation resulted in strong docking scores between YfhL and *Av*NifH, with free binding energies ranging from −12.5 to −17.3 kcal/mol and a minimum distance of 7.2 Å between the [Fe_4_S_4_] cluster of *Av*NifH and [Fe_4_S_4_] cluster 1 of YfhL achieved at a binding energy of −13.3 kcal/mol (Fig. 3, A to C). Motivated by this observation, we overexpressed YfhL in *E. coli* and subsequently isolated this protein (fig. S3) for the spectroscopic and enzymatic investigations of its interaction with *Av*NifH. EPR analysis revealed an electron transfer from the reduced YfhL to the oxidized *Av*NifH*^Ec^*, which was reflected by the appearance of the *Av*NifH*^Ec^*-associated [Fe_4_S_4_]^+^ signal concomitant with the attenuation of the YfhL-associated [Fe_4_S_4_]^+^ signal upon incubation (Fig. 3D). Frequency-selective pulse ^1^H NMR analysis provided further evidence for the competence of YfhL as an electron donor to drive the ATP-dependent N_2_ reduction by the *Av*NifH*^Ec^*/*Av*NifEN^*Ec*-L^ system, showing a 75% higher activity of NH_4_^+^ formation upon substitution of 0.3 mM dithionite with an equivalent amount of pre-reduced YfhL and a further increase in NH_4_^+^ formation with increasing amounts of pre-reduced YfhL (Fig. 3E).

### In vivo N_2_ reduction by the nitrogenase analog comprising NifH/NifEN

**Fig. 4.**
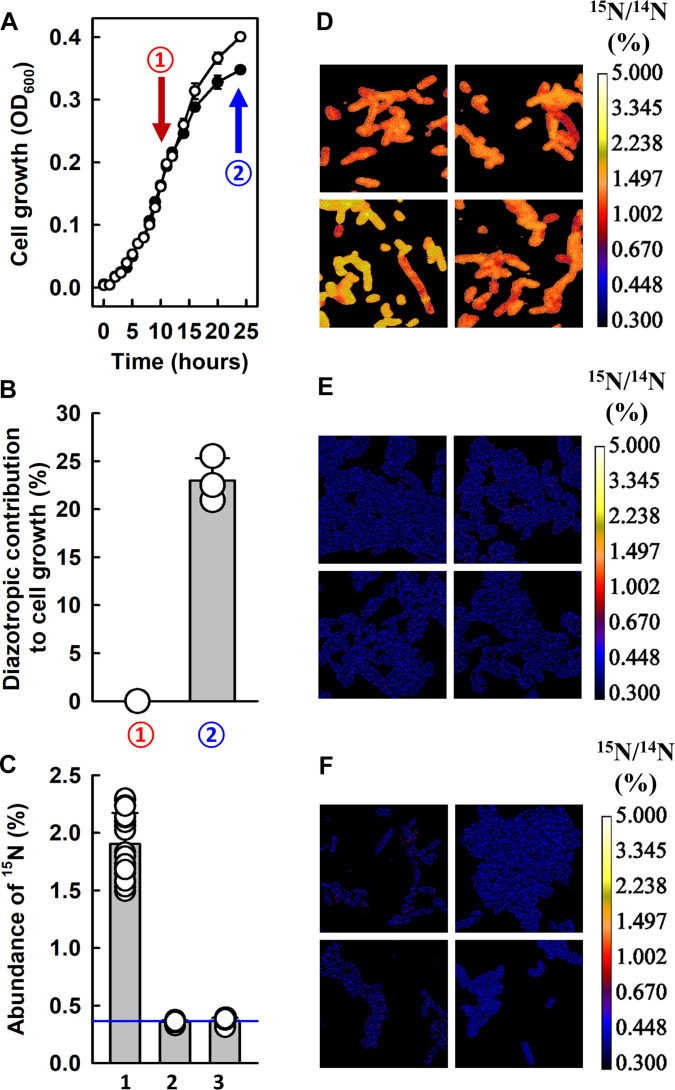
Diazotrophic nitrogen assimilation by *E. coli* co-expressing *Av*NifH*^Ec^* and *Av*NifEN^*Ec*-L^. (**A**) Cell growth of *E. coli* strain YM578EE in a medium containing a limited amount of ammonia (2 mM) in the presence 100% N_2_ (open circles) or Ar (solid circles). Nitrogenase expression was induced when the cell growth reached ~50% of the maximum cell density (or ~50% consumption of the externally supplied ammonia in the medium), 10 hours post-inoculation (①), followed by continued cell growth until 24 hours post-inoculation (②). Data are expressed as means ± SEM (*n* = 3). (**B**) Diazotrophic contribution to the cell growth of YM578EE, showing a 23.0 ± 2.3% gain of cell density 14 hours post-induction. Diazotrophic contribution (expressed as a percentage) at the beginning (①) or the end (②) of isopropyl β-d-thiogalactopyranoside (IPTG) induction was calculated by dividing the difference between cell densities under N_2_ (A, open circle) and Ar (A, closed circle) by the cell density under Ar [(A), closed circle] at 10 or 24 hours post-inoculation. Data are expressed at means ± SD (*n* = 3). (**C**) Statistical analysis of secondary ion images derived from nanoSIMS experiments of three *E. coli* strains (**D** to **F**): (1) YM578EE (*yfhL*-replete) expressing *Av*NifH*^Ec^* and *Av*NifEN^*Ec*-L^ under 100% ^15^N_2_ [shown in (D)], (2) MY21 (nitrogenase-free) grown under 100% ^15^N_2_ [shown in (E)], and (3) YM634EE (*yfhL*-deficient) expressing *Av*NifH*^Ec^* and *Av*NifEN^*Ec*-L^ under 100% ^15^N_2_ [shown in (F)]. The ^15^N abundance [C, (1) to (3)] of each strain was calculated on the basis of data collected in six different regions of interest of the nanoSIMS images of four independent samples [in (D) to (F)]. The average values of ^15^N abundance (or ^15^N/^14^N) are (1) 1.90 ± 0.27%, (2) 0.36 ± 0.01%, and (3) 0.37 ± 0.03%. Given the natural ^15^N abundance (^15^N/^14^N) of 0.37% [indicated by a blue line in (C)], the level of ^15^N enrichment for each strain is (1) 5.1-fold, (2) 1-fold, and (3) 1-fold.

The outcome of the in vitro studies of YfhL provided strong support for the feasibility for our *E. coli* expression strain to use YfhL to drive the in vivo N_2_ reduction by the two-component nitrogenase analog comprising *Av*NifH*^Ec^* and *Av*NifEN^*Ec*-L^. To test this possibility, we cultivated the *E. coli* strain (YM578EE) with a limited amount of externally supplied NH_4_^+^ (2 mM), induced co-expression of *Av*NifH*^Ec^* and *Av*NifEN^*Ec*-L^ with isopropyl β-d-thiogalactopyranoside (IPTG) (0.5 mM) upon ~50% consumption of NH_4_^+^ in the growth medium, and continued to monitor cell growth under N_2_ or Ar afterward. Such a procedure should permit a preinduction accumulation of cell mass and biosynthetic machineries in preparation for the post-induction synthesis of nitrogenase, with the latter initially supported by the remaining NH_4_^+^ in the growth medium but eventually allowing the cells to fix N_2_ on their own. Consistent with this argument, a comparison of the post-induction growth of *E. coli* strain YM578EE under N_2_ and Ar revealed that the former clearly outpaced the latter (Fig. 4A), resulting in an increased gain of cell yield by 23.0% at 14 hours post-induction (Fig. 4B).

The in vivo N_2_-fixing activity of the *E. coli* expression strain, as well as the ability of YfhL to support this activity as a physiological electron donor, was further demonstrated by nanoscale secondary ion mass spectrometry (nanoSIMS) analyses that compared the level of N assimilation of the nitrogenase-expressing strain (YM578EE) with that of the nitrogenase-free control (MY21) and with that of the nitrogenase-expressing strain wherein *yfhL* was deleted (YM634EE). For this line of experiments, samples were prepared by a protocol similar to that used for the growth experiments, except for the use of ^15^N_2_ instead of ^14^N_2_. Subsequently, the cells were harvested, fixed, and dried for nanoSIMS analyses by a CAMECA nanoSIMS 50L instrument. A detailed comparison of the secondary ion images, accomplished through statistical analyses of six different regions of interest of the images of four individual samples, revealed a clear distinction in the levels of ^15^N enrichment of the ^15^N_2_-grown YM578EE and MY21. Specifically, YM578EE displayed an average ^15^N/^14^N ratio of 1.90 ± 0.27% (Fig. 4C, 1; Fig. 4D), 5.1 times higher than the natural abundance ^15^N/^14^N ratio of 0.37%, whereas MY21 displayed an average ^15^N/^14^N ratio of 0.36 ± 0.01% (Fig. 4C, 2; Fig. 4E), nearly identical to the background ^15^N/^14^N ratio of 0.37%. Moreover, YM634EE, a YM578EE-derived, *yfhL*-deletion strain, was unable to accumulate ^15^N beyond the ^15^N/^14^N background (Fig. 4C, 3; Fig. 4F). It is important to note that the *Av*NifEN^*Ec*-L^ protein co-expressed with *Av*NifH*^Ec^* in YM634EE was L-cluster replete and capable of in vitro N_2_ reduction when combined with *Av*NifH*^Ec^*, ATP, and dithionite (fig. S4, A to C). As such, the lack of in vivo N_2_-reducing activity in the case of YM634EE was clearly associated with the absence of YfhL as an appropriate, low-potential electron donor for this reactivity.

It is worth noting that the endogenous flavodoxin FldA expressed along with its reductase, YdbK, in *E. coli* was shown to increase the in vivo acetylene reduction activity over a 24-hour time period (*39*). However, like other flavodoxins and ferredoxins without two FeS centers, FldA has a more positive reduction potential [−433 mV; (*40*)] than the low-potential cluster in YfhL (−675 mV) and, therefore, is unlikely to function as an electron donor for NifH in our system. This argument is further supported by the inability of the *yfhL*-deficient *E. coli* strain YM634EE to perform N_2_ fixation despite the presence of the endogenous FldA in this strain (Fig. 4F). Compared to N_2_, C_2_H_2_ is a much easier substrate that can be reduced at a more positive potential adopted by FldA. Additionally, the whole-cell C_2_H_2_-reducing activity cannot be equated with the ability of the expression strain to perform N_2_ fixation, as C_2_H_2_ reduction can be readily accomplished by simple FeS proteins or even FeS clusters (*41*). As such, it is likely that the nitrogenase reaction requires a low-potential, two-cluster ferredoxin like YfhL as an electron donor instead of flavodoxins that have more positive redox potentials.

## DISCUSSION

### Implications of the N_2_-reducing activity of a simplified nitrogenase analog

The ability of *Av*NifEN^*Ec*-L^/*Av*NifH*^Ec^* to function as a nitrogenase analog in vivo and endow *E. coli* with an “assisted” diazotrophic capacity offers strong support to our previous proposal that the L-cluster–bound NifEN represents a prototype ancient nitrogenase that gave rise to the modern-day nitrogenase (*29*). Supported by biochemical and phylogenetic evidence and backed further by ecological and mechanistic considerations, this proposal centers on the sequence and structural conservation of NifEN/NifDK and their active-site L/M-clusters and posits a concomitant evolution of the protein scaffold and the active site that transforms a low-efficiency, evolutionary predecessor (carrying a surface-exposed, all-Fe cofactor) to a high-efficiency, modern-day nitrogenase (containing a well-enclosed, Mo/homocitrate-replete cofactor). Evolution of the active site of nitrogenase features a hypothesized theme of breaking the three-fold symmetry of the ancestral, all-Fe cofactor (*29*), which would enable the functional distinction of the three seemingly equivalent belt-sulfur locations and an effective utilization of all three positions for a plausible, stepwise mechanism of N_2_ reduction (*42*). The proposal of such an asymmetric reaction site of the present-day nitrogenase has gained strong support from our recent studies, which pointed to the capture of distinct N_2_ species at different belt-S sites under electron- and sulfur-depleted conditions (*43*, *44*). As such, a comparison of the reactivity achieved by the asymmetric reaction site of the extant nitrogenase (i.e., NifDK) with that achieved by the symmetric reaction site of the prototype nitrogenase (i.e., NifEN) could provide crucial insights into the evolution and mechanism of this important metalloenzyme.

In a practical vein, the successful co-expression of NifEN and NifH as a two-component nitrogenase analog in *E. coli* marks an important step toward achieving true diazotrophy in a non-diazotrophic model organism. Such a simplified N_2_-reducing system eliminates the need to (i) express *nifD* and *nifK* and (ii) mature the L-cluster into an M-cluster on NifEN and subsequently transfer the M-cluster to apo-NifDK, thereby substantially reducing the biosynthetic burden to the host. Additionally, the homocitrate-free composition of the L-cluster omits the requirement to synthesize this organic compound via condensation of acetyl–coenzyme A and 2-oxoglutarate, thereby eliminating the need to consume two key metabolites in the TCA cycle of the host. Last, the identification of YfhL as a suitable in vivo electron donor suggests the possibility to further reduce the biosynthetic burden for the host by “borrowing” its endogenous components for the heterologous synthesis of nitrogenase. Characterized by these desirable features for the expression host, this previously unidentified nitrogenase system represents a viable platform for further optimization via improvement of the cluster occupancy and/or the catalytic efficiency of NifEN. With respect to the former, the “upstream” production of L-clusters on NifB, as well as the transfer of L-clusters from NifB to NifEN, could be tackled to achieve a full complement of L-clusters in NifEN. With respect to the latter, attempts could be made to generate a functional equivalent of NifDK via engineering of the L/O-cluster sites in NifEN toward those resembling the M/P-cluster sites in NifDK while maintaining the biosynthetic ability of NifEN to mature the L-cluster into an M-cluster. The feasibility of this line of work was illustrated by our previous success in the heterologous expression of a NifEN variant with an M-cluster “locked” at its binding site (*30*). It is conceivable, therefore, that the engineering efforts of NifEN could result in a self-assembling NifDK equivalent with a high efficiency of N_2_ reduction, which could facilitate transgenic expression of nitrogenase in higher organisms or enable development of nitrogenase-based biotechnological applications in the future.

## MATERIALS AND METHODS

All chemicals were purchased from Sigma-Aldrich (St. Louis, MO) and Thermo Fisher Scientific (Waltham, MA), unless specified otherwise. All experiments were conducted in a glove box or on a Schlenk line under an Ar atmosphere, with an O_2_ concentration of <3 ppm.

### Strain construction

The genes encoding the *A. vinelandii* NifH (with or without an N-terminal polyhistidine tag), NifM, NifE (with an N-terminal polyhistidine tag), and NifN proteins and the *M. acetivorans* NifS3, NifU3, and NifB proteins were codon optimized for *E. coli* expression, synthesized, and cloned into pCDFDuet-1 (carrying the *Ma*_*nifS3*,*U3*,*B* and *Av*_*nifE*,*N genes*), pRSFDuet-1 (carrying the *Av*_*nifH*,*M* genes), respectively (GenScript, Piscataway, NJ). Subsequently, these constructs were transformed into *E. coli* strain MY21, which was derived from *E. coli* strain BL21(DE3) but contained a deletion of *iscR*, the gene encoding the FeS cluster regulator, in the genome. This procedure resulted in *E. coli* strains expressing a nitrogenase analog comprising (i) a non-tagged, [Fe_4_S_4_] cluster–containing *Av*NifH*^Ec^* and a His-tagged, O-cluster ([Fe_4_S_4_])– and L-cluster ([Fe_8_S_9_C])–containing *Av*NifEN^*Ec*-L^ (strain YM578EE); or (ii) a His-tagged, [Fe_4_S_4_] cluster–containing *Av*NifH*^Ec^* and a His-tagged, O- and L-cluster–containing *Av*NifEN^*Ec*-L^ (strain YM586EE) upon induction with IPTG.

Using the no-SCAR (Scarless Cas9 Assisted Recombineering) method (*45*), *E. coli* strain MY25 was constructed by deleting *yfhL*, the gene encoding a low-potential, two–[Fe_4_S_4_] cluster–containing ferredoxin (YfhL), from the genome of MY21. The plasmids pCas9-CR4, pKDsgRNA-ack, and pKDsgRNA-p15 were a gift from K. Prather (Addgene, plasmid nos. 62655, 62656, and 62654; http://n2t.net/addgene:62655, http://n2t.net/addgene:62656, and http://n2t.net/addgene:62654;RRID: Addgene_62655, Addgene_62656, and Addgene_62654). The same pCDFDuet-1– and pRSFDuet-1–derived constructs (see above) were then transformed into MY25, resulting in an *E. coli* strain expressing a nitrogenase analog comprising a His-tagged, [Fe_4_S_4_] cluster–containing *Av*NifH*^Ec^* and a His-tagged, O- and L-cluster–containing *Av*NifEN^*Ec*-L^ (strain YM634EE) in a *yfhL*-deletion background upon induction with IPTG.

Other than the strains described above, a previously reported *E. coli* strain (YM253EE) expressing a His-tagged, O-cluster–containing yet L-cluster–deficient *Av*NifEN^*Ec*-apo^ was also used for the in vitro reconstitution with the solvent-extracted L-cluster, which resulted in an O- and L-cluster–replete *Av*NifEN^recon^ (*30*). Additionally, the gene encoding the *E. coli* YfhL protein was synthesized and cloned into pET-24a(+) (GenScript, Piscataway, NJ), followed by transformation of this construct into MY21, resulting in an *E. coli* strain overexpressing YfhL upon induction with IPTG (strain YM646EE).

### Cell growth and protein purification

*E. coli* strains YM578EE, YM586EE, YM634EE, YM253EE, and YM646EE were grown in 10-liter batches in LB medium (Difco) supplemented with 50 mM MOPS/NaOH (pH 7.4); 25 mM glucose; 2 mM ferric ammonium citrate; and either chloramphenicol (20 mg/liter), kanamycin (19 mg/liter), and streptomycin (26 mg/liter; for YM578EE, YM586EE, and YM634EE) or chloramphenicol (25 mg/liter) and ampicillin (50 mg/liter) (for YM253EE and YM646EE) in a BIOFLO 415 fermenter (New Brunswick Scientific) at 37°C with agitation at 200 rpm and airflow of 10 liters/min. When the optical density at 600 nm (OD_600_) reached 0.5, the airflow was terminated, and the fermenter was purged with N_2_ (ultrahigh purity) at a rate of 1.5 liters/min; additionally, the temperature was lowered to 24°C. Once the temperature reached 24°C, 25 mM sodium fumarate and 2 mM cysteine were added, and the expression of nitrogenase was induced by the addition of 250 μM IPTG. Protein expression was allowed to continue for 16 hours, followed by harvesting of the cells by centrifugation using a Thermo Fisher Scientific Legend XTR centrifuge. The heterologously expressed, His-tagged *Av*NifEN^*Ec*-L^, *Av*NifEN^*Ec*-apo^, and YfhL proteins were purified from strain YM578EE, YM253EE, and YM646EE, respectively, by IMAC using a method adapted from the purification of the His-tagged nitrogenase proteins from *A. vinelandii* (*46*), whereas the heterologously co-expressed, His-tagged *Av*NifH*^Ec^* and His-tagged *Av*NifEN^*Ec*-L^ were co-purified at a molar ratio of ~1:1 from strain YM586EE or YM634EE by IMAC, which was used as is or subjected to SEC to further separate *Av*NifH*^Ec^* and *Av*NifEN^*Ec*-L^ as described earlier (*46*).

*A. vinelandii* strains DJ1162, DJ1141, DJ1143, and DJ1041 expressing His-tagged *Av*NifH, *Av*NifDK, *Av*NifDK^apo^, and *Av*NifEN^L^ (*34*, *47*), respectively, were grown in 180-liter batches in Burke’s minimal medium supplemented with 2 mM ammonium acetate in a 200-liter fermenter (New Brunswick Scientific) at 30°C with agitation at 100 rpm and airflow of 30 liters/min. Cell growth was monitored at OD_436_ using a Spectronic 20 Genesys spectrometer (Spectronic Instruments) and, upon depletion of ammonia, cells were derepressed for 3 hours before harvesting by a flow-through centrifugal harvester (Cepa). Published methods were used to purify His-tagged *Av*NifH, *Av*NifDK, *Av*NifDK^apo^, and *Av*NifEN^L^ (*34*, *47*).

### Metal analysis

The metal contents of *Av*NifH, *Av*NifH*^Ec^*, *Av*NifEN^L^, and *Av*NifDK^*Ec*-L^ were determined by inductively coupled plasma optical emission spectroscopy (ICP-OES) using the iCAP7000 ICP-OES machine (Thermo Scientific Scientific). Calibration of the equipment was performed by using standard solutions made via dilution of a stock solution of elemental Fe (1 mg/ml). The protein sample was first mixed with 100 μl of concentrated sulfuric acid (H_2_SO_4_) and 100 μl of concentrated nitric acid (HNO_3_) and subsequently heated for 30 min at 250°C. Such a procedure was repeated until the solution became colorless, followed by cooling of the solution to room temperature, and dilution of the solution to a total volume of 7.5 ml with 2% HNO_3_ before metal analysis.

### Substrate reduction assays

The C_2_H_2_ reduction assays were performed at 30°C in 9.5-ml vials fitted with rubber serum stoppers and metal caps (DWK Life Science, Millville, NJ). The assay that tested the specific activity of *Av*NifH*^Ec^* contained, in a total volume of 1 ml, 25 mM tris-HCl (pH 8.0), 2.5 mM ATP, 5.0 mM MgCl_2_, 30 mM creatine phosphate, 0.125 mg of creatine phosphokinase, and 20 mM sodium dithionite (Na_2_S_2_O_4_); additionally, it contained 0.1-atm C_2_H_2_ and 0.9-atm Ar in the headspace. The reaction was initiated by the addition of 2.4 mg of *Av*NifDK and 0.36 mg of *Av*NifH*^Ec^*, followed by incubation at 30°C for 10 min, quenching with EDTA, and analysis of product formation. The reaction that tested the specific activity of *Av*NifEN^*Ec*-L^ contained, in a total volume of 1 ml, 25 mM tris-HCl (pH 8.0), 2.5 mM ATP, 5.0 mM MgCl_2_, 30 mM creatine phosphate, 0.125 mg of creatine phosphokinase, and 0.5 mM Na_2_S_2_O_4_; additionally, it contained 0.5-atm C_2_H_2_ and 0.5-atm Ar in the headspace. The reaction was initiated by the addition of 0.15 mg of *Av*NifEN^*Ec*-L^ and 1.75 mg of *Av*NifH, followed by incubation at 30°C for 20 min, quenching with EDTA, and analysis of product formation. To detect C_2_H_4_ as a product of C_2_H_2_-reduction, 250 μl of the headspace was injected into a Gas Chromatography-Flame Ionization Detector (GC-FID) (SRI Instruments, Torrance CA) equipped with a packed Poropak N column (Restek, Bellefonte, PA). Calibration was achieved by injecting 15-ppm C_2_H_4_ gas standard under the same conditions.

### M-cluster maturation assays

Each maturation assay contained, in a total volume of 0.9 ml, 25 mM tris-HCl (pH 8.0), 0.4 mg of the P-cluster–containing yet M-cluster–deficient *Av*NifDK^apo^ [isolated from *A. vinelandii* DJ1143; (*25*, *47*)], 1.2 mg of *Av*NifH, 1.0 mg of the O- and L-cluster–containing *Av*NifEN^*Ec*-L^ or *Av*NifEN^L^ [isolated from *A. vinelandii* strain DJ1041; (*34*)], 0.4 mM homocitrate, 0.4 mM Na_2_MoO_4_, 2.4 mM ATP, 4.8 mM MgCl_2_, 30 mM creatine phosphate, 24 U of creatine phosphokinase, and 20 mM Na_2_S_2_O_4_. Additionally, each assay contained 1.0-atm Ar in the headspace. The reaction was incubated at 30°C for 60 min and subsequently split into triplicates in three 9.5-ml vials, each containing 1.05 mg of *Av*NifH, 25 mM tris-HCl (pH 8.0), 2.5 mM ATP, 5.0 mM MgC1_2_, 30 mM creatine phosphate, 0.125 mg of creatine phosphokinase, and 20 mM Na_2_S_2_O_4_ in a total volume of 0.7 ml; additionally, it contained 0.1-atm C_2_H_2_ and 0.9-atm Ar in the headspace. The reaction mixture was then incubated at 30°C for 10 min and analyzed for product formation as described above.

### EPR experiments

Individual EPR samples were prepared in a Vacuum Atmospheres glove box filled with Ar and operated at <3-ppm O_2_ and flash frozen in liquid nitrogen before analysis. The reduced (Red) samples contained 10% (v/v) glycerol, 250 mM imidazole, 2 mM Na_2_S_2_O_4_, and 25 mM tris-HCl (pH 8.0); the oxidized (Ox) samples were prepared by incubating the reduced samples with excess IDS for 5 min; and the super-reduced (SR) samples were prepared by adding excess europium(II) EGTA (Eu^II^-EGTA). The concentration of the reduced or oxidized *Av*NifH*^Ec^*, *Av*NifEN^*Ec*-L^, or YfhL sample was 15 mg/ml, and the concentration of the super-reduced *Av*NifH*^Ec^* sample was 10 mg/ml. EPR data were acquired using an ESP 300E spectrophotometer (Bruker) interfaced with an ESR-9002 liquid-helium continuous-flow cryostat (Oxford Instruments), with a microwave power of 5 mW, a gain of 5×10^4^, a modulation frequency of 100 kHz, and a modulation amplitude of 5 G. Eight scans of perpendicular-mode EPR spectra were recorded for each sample at 10 K (for the reduced *Av*NifEN^*Ec*-L^ sample and the reduced or oxidized *Av*NifH*^Ec^* and YfhL samples) and 15 K (for the oxidized *Av*NifEN^*Ec*-L^ sample), respectively, using a microwave frequency of 9.62 GHz; and 16 scans of parallel-mode EPR spectra were recorded for each sample at 10 K (for the super-reduced *Av*NifH*^Ec^* sample), using a microwave frequency of 9.38 GHz.

To probe the transfer of electrons from YfhL to *Av*NifH*^Ec^*, the reduced YfhL protein was mixed with the oxidized *Av*NifH*^Ec^* protein at a molar ratio of 1:2, resulting in a total protein concentration of 25 mg/ml. The mixture was then incubated with stirring for 1 hour at room temperature under an Ar atmosphere. A 300-μl aliquot was taken from the mixture immediately after the mixing of the two proteins (the “0-hour” sample) and 1 hour into the incubation of the mixture (the “1-hour” sample), followed by an immediate transfer of each aliquot to an EPR tube, wherein the sample was flash frozen for the EPR analysis as described above.

### Cell growth experiments

A 10-ml culture of *E. coli* strain YM578EE was grown in LB medium (Difco) supplemented with 50 mM MOPS/NaOH (pH 7.4), 25 mM glucose, 2 mM ferric ammonium citrate, chloramphenicol (20 mg/liter), kanamycin (19 mg/liter), and streptomycin (26 mg/liter) in a 50-ml conical tube at 37°C with agitation at 200 rpm overnight. Subsequently, two 1-ml aliquots of this overnight culture were transferred to two 1.5-ml microtubes and centrifuged at 10,000 rpm for 4 min. After carefully removing the supernatant, each cell pellet was washed and resuspended in 0.2 ml of supplemented M9 medium that contained 0.4% glucose, 2 mM MgSO_4_, 0.1 mM CaCl_2_, Na_2_HPO_4_ (12.8 mg/ml), KH_2_PO_4_ (3 mg/ml), NaCl (0.5 mg/ml), 2 mM NH_4_Cl, FeCl_3_ (0.054 mg/ml), and 1 mM cysteine. The resuspended cells derived from each pellet were inoculated into 100 ml of supplemented M9 medium containing chloramphenicol (20 mg/liter), kanamycin (19 mg/liter), and streptomycin (26 mg/liter) in a 250-ml screw-capped Erlenmeyer flask with its side-arm capped by a septum. The two cultures were initially grown aerobically with a loose cap on at 37°C and agitation at 200 rpm, with 200 μl of each culture removed at a time interval of 1 hour via the side-arm septum using a sterile syringe for the determination of cell density (at OD_600_) and ammonia concentration (see below). After 10 hours of aerobic cell growth (when OD_600_ reached ~0.17), the gas phases of these flasks were exchanged with either 100% Ar or 100% N_2_ by sparging under the respective gases for 5 min, followed by addition of 0.5 mM IPTG to each culture for the induction of protein expression. The cultures were then grown until a total of 24 hours was reached from the time point of inoculation, with a continuous removal of 200-μl culture at a time interval of 1 hour.

The concentration of ammonia in each 200-μl culture aliquot was determined using an *o*-phthalaldehyde (OPA) reagent–based method. Briefly, the 200-μl culture aliquot was first centrifuged at 10,000 rpm for 4 min, followed by addition of 100 μl of the supernatant to a solution that contained, in a total volume of 1 ml, 10 mM OPA and 2.5 mM 2-mercaptoethanol in a 50 mM potassium phosphate buffer (pH 7.8). The mixture was then incubated at room temperature for 3 hours, followed by pipetting onto a 96-well proxiplate (PerkinElmer, MA) and measurement using a microplate reader (Spark, Tecan, CA) with the excitation wavelength set at 360 nm and the emission wavelength set at 425 nm.

### NanoSIMS analysis

*E. coli* strains YM578EE (a strain co-expressing *Av*NifH*^Ec^* and *Av*NifEN^*Ec*-L^ in a *yfhL*-replete background), YM634EE (a strain co-expressing *Av*NifH*^Ec^* and *Av*NifEN^*Ec*-L^ in a *yfhL*-deletion background), and MY21 (a negative control strain that does not express any nitrogenase protein) were grown in 100 ml of supplemented M9 medium (see above for composition), with the YM578EE culture containing chloramphenicol (20 mg/liter), kanamycin (19 mg/liter), and streptomycin (26 mg/liter) in 250-ml screw-capped Erlenmeyer flasks with septum-capped side arms for 10 hours before the gas phases were exchanged into 100% ^15^N_2_. Subsequently, 0.5 mM IPTG was added to the YM578EE or YM634EE culture, followed by continued cell growth for another 12 hours before nanoSIMS analysis.

For nanoSIMS analysis, a 250-μl aliquot of each culture (diluted to the same OD) was pipetted onto a 7 mm–by–7 mm silicon square dice cut from wafers (UniversityWafer, Boston, MA) with a diameter of 2.5 cm. The samples were subsequently (i) fixed with a phosphate-buffered saline (PBS) solution containing 4% formaldehyde for 1 hour at room temperature; (ii) washed sequentially with PBS, 1:1 PBS/ethanol, and ethanol; and (iii) dried on the wafer (*48*). The secondary ion (^14^N^12^C^−^ and ^15^N^12^C^−^) and secondary electron images were acquired with the CAMECA NanoSIMS 50L ion microprobe at Caltech (Pasadena, CA). A +8-keV primary Cs^+^ beam of ∼1 pA was used to raster the samples in 20 μm–by–20 μm areas. Secondary ion (^14^N^12^C^−^ and ^15^N^12^C^−^) images of −8 keV were collected simultaneously with electron multiplier detectors. The interferences (e.g., ^13^C^13^C^−^ to ^14^N^12^C^−^; and ^14^N^13^C^−^ to ^15^N^12^C^−^) were fully removed from the masses of interest under the high mass resolution conditions of the mass spectrometer. Ion images of 512 by 512 pixels were processed with the L’image software (http://limagesoftware.net/).

### Frequency-selective pulse NMR analysis

To assess ATP-dependent reduction of N_2_ by *Av*NifH*^Ec^* and *Av*NifEN^*Ec*-L^, each assay contained, in a total volume of 1.3 ml, 25 mM tris-HCl (pH 8.0), 2.5 mM ATP, 5.0 mM MgCl_2_, 30 mM creatine phosphate, 0.125 mg of creatine phosphokinase, 20 mM Na_2_S_2_O_4_, and either (i) 1.4 mg of *Av*NifEN^*Ec*-L^ and 3.15 mg of *Av*NifH*^Ec^* (individually purified from YM578EE and mixed at a molar ratio of 1:8) or (ii) 1.7 mg of *Av*NifEN^*Ec*-L^/*Av*NifH*^Ec^* (co-purified from YM586EE or YM634EE at a molar ratio of ~1:1). Each negative control contained, in a total volume of 1.3 ml, 25 mM tris-HCl (pH 8.0), 20 mM Na_2_S_2_O_4_, and (i) 1.4 mg of *Av*NifEN^*Ec*-apo^, 3.15 mg of *Av*NifH*^Ec^*, 5.0 mM MgCl_2_, 30 mM creatine phosphate, and 0.125 mg of creatine phosphokinase; (ii) 1.4 mg of *Av*NifEN^*Ec*-L^, 3.15 mg of *Av*NifH^*Ec*-apo^, 5.0 mM MgCl_2_, 30 mM creatine phosphate, and 0.125 mg of creatine phosphokinase; or (iii) 1.4 mg of *Av*NifEN^*Ec*-L^ and 3.15 mg of *Av*NifH*^Ec^*. *Av*NifH^*Ec*-apo^ was prepared by chelation of the [Fe_4_S_4_] cluster with bathophenanthroline as described previously (*20*).

To illustrate the role of YfhL as an electron donor for N_2_ reduction by *Av*NifH*^Ec^*/*Av*NifEN^*Ec*-L^, each assay contained, in a total volume of 1.3 ml, 25 mM tris-HCl (pH 8.0), 2.5 mM ATP, 5.0 mM MgCl_2_, 30 mM creatine phosphate, 0.125 mg of creatine phosphokinase, 1.4 mg of *Av*NifEN^*Ec*-L^, 3.15 mg of *Av*NifH*^Ec^*, and (i) 0.3 mM Na_2_S_2_O_4_, (ii) 0.3 mM (4 mg) YfhL, (iii) 0.6 mM (8 mg) YfhL, or (iv) 0.9 mM (12 mg) YfhL.

Each mixture (above) was incubated at 30°C for 60 min under a gas atmosphere of 100% ^15^N_2_, followed by transfer of the mixture to a 1.5-ml tube containing a Microcon centrifugal filter with a Molecular Weight Cut-Off (MWCO) of 10 kDa (Millipore). Subsequently, the proteins were removed from the mixture by centrifugation at 10,000*g* for 20 min, and 0.5 ml of the flow-through (i.e., the protein-removed mixture) was combined with 0.05 ml of 1 M H_2_SO_4_ and 0.025 ml of CD_3_CN as a lock agent. The ^1^H NMR spectra were recorded using a Bruker AVANCE600 spectrometer equipped with a CBBFO cryoprobe. Water suppression was used, and spectra were referenced by setting the residual CH_3_CN signal to 2.06 ppm (*49*). A total of 4096 scans were recorded per sample, with an acquisition time of 1.5 s and a relaxation delay of 5 s.

### Docking calculations

The feasibility of whether the two [Fe_4_S_4_] cluster–containing ferredoxin, YfhL, from *E. coli* could form physiologically viable complexes with the NifH protein from *A. vinelandi* (*Av*NifH) was evaluated by using a combined docking/energy evaluation approach as described previously (*38*). To this end, the crystal structures of YfhL [Protein Data Bank (PDB) entry 2ZVS] and *Av*NifH (PDB entry 2NIP) were used for docking calculations with the ClusPro server (*50*–*53*), followed by determination of the interaction energies for all 54 docking solutions with FoldX (*54*–*56*) in a process automated via Python/Shell scripting. Of the 54 solutions, the 8 energetically most favorable solutions (with binding energies < −12 kcal/mol) were further analyzed on the basis of the distance between the [Fe_4_S_4_] cluster of *Av*NifH and the proximally located [Fe_4_S_4_] cluster (i.e., [Fe_4_S_4_] cluster 1) of YfhL. A minimum distance of 7.2 Å was determined for the distance between the [Fe_4_S_4_] cluster of *Av*NifH and [Fe_4_S_4_] cluster 1 of YfhL at a binding energy of −13.3 kcal/mol.
