# Supplementary material for: Heterologous synthesis of a simplified nitrogenase analog in Escherichia coli
Source: Sci Adv. 2025 May 2;11(18):eadw6785. doi: 10.1126/sciadv.adw6785 (PMC12047441; doi:10.1126/sciadv.adw6785)
Supplement: Supplementary file 1 — Figs. S1 to S4 Tables S1 and S2 [file sciadv.adw6785_sm.pdf]

Science Advances
AAAS

Supplementary Materials for

Heterologous synthesis of a simplified nitrogenase analog in *Escherichia coli*

Yiling A. Liu *et al.*

Corresponding author: Markus W. Ribbe, mribbe@uci.edu; Yilin Hu, yilinh@uci.edu

Sci. Adv. **11**, eadw6785 (2025)
DOI: 10.1126/sciadv.adw6785

This PDF file includes:

Figs. S1 to S4
Tables S1 and S2

Supplementary Figures

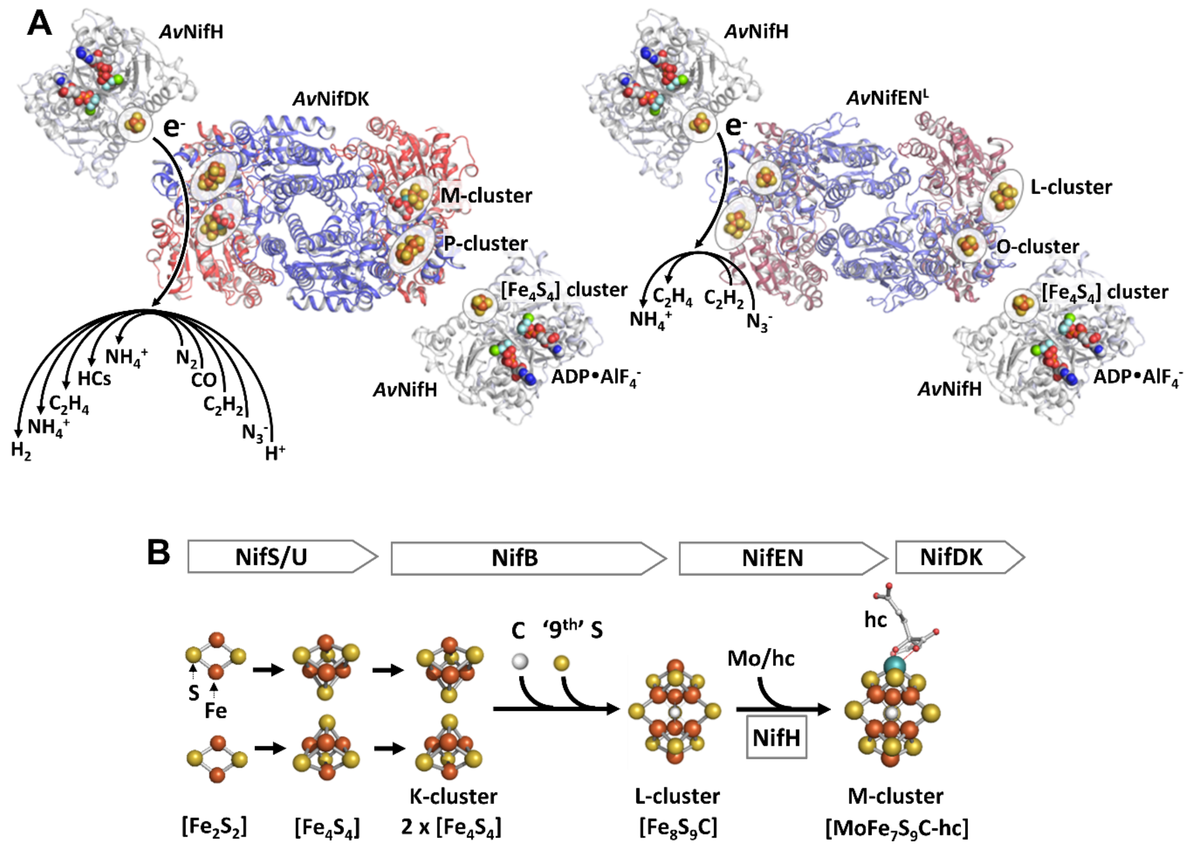

fig. S1. Nitrogenase catalysis and assembly. (**A**) Structures of the *Azotobacter vinelandii* Mo-nitrogenase comprising the reductase component (*Av*NifH) and the catalytic component (*Av*NifDK) (*left*) and a simplified nitrogenase analog comprising the reductase component (*Av*NifH) and the cofactor maturase (*Av*NifEN^L) (*right*). Catalysis by the Mo-nitrogenase (*left*) involves transfer of the electrons from the [Fe_4S_4] cluster of *Av*NifH, through the P-cluster ([Fe_8S_7]), to the M-cluster ([(*R*-homocitrate)$MoFe_7S_9C$]) of *Av*NifDK, where substrate reduction occurs. By analogy, catalysis by the nitrogenase analog (*right*) presumably involves transfer of the electrons from the [Fe_4S_4] cluster of *Av*NifH, through the O-cluster ([Fe_4S_4]), to the L-cluster ([Fe_8S_9C]) of *Av*NifEN^L. The two subunits of *Av*NifH are colored light and blue gray, respectively; the α- and β-subunits of *Av*NifDK are colored red and blue, respectively; and the α- and β-subunits of *Av*NifEN^L are colored light red and light blue, respectively. The atoms are colored as follows: Fe, orange; S, yellow; Mo, cyan; C, light grey; Mg, green; C, light grey; N, blue; O, red; P, dark orange; Al, dark gray; F, light blue. PyMOL was used to generate this figure, using PDB entries 1N2C, 3U7Q and 3PDI. (**B**) Assembly of the M-cluster of the *A. vinelandii* Mo-nitrogenase begins with the formation of [Fe_4S_4] clusters by NifS/U, followed by transfer of a K-cluster (2x[Fe_4S_4]) to NifB for the radical SAM-dependent coupling/rearrangement into an L-cluster ([Fe_8S_9C]) concomitant with the insertion of an interstitial C and a '9th' belt-S. Subsequently, the L-cluster is transferred to NifEN and matured into an M-cluster via NifH-mediated insertion of Mo and homocitrate (hc) prior to delivery of the M-cluster to its target location in NifDK.

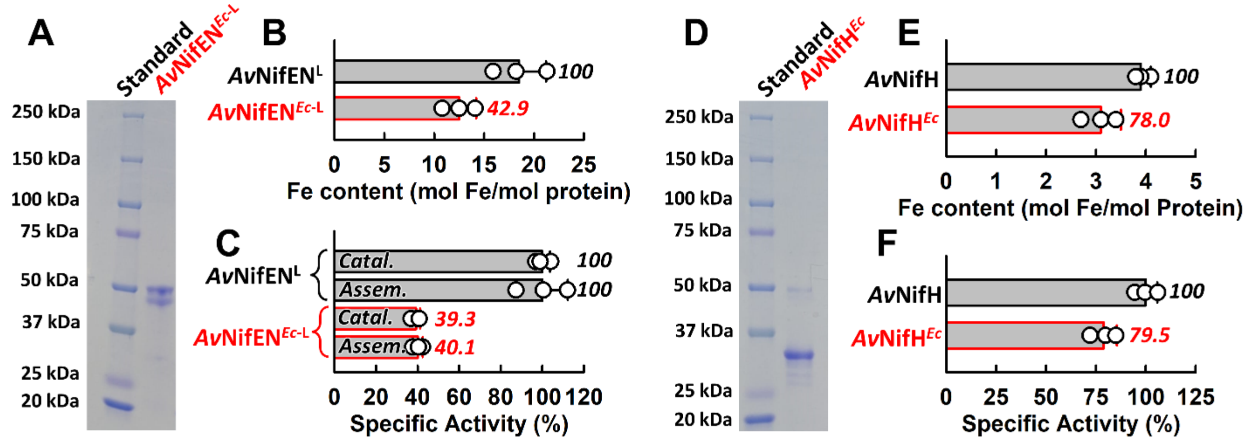

fig. S2. Biochemical characterization of *Av*NifEN$^{Ec\text{-}L}$ and *Av*NifH^{Ec}. (**A**) SDS-PAGE of the heterologously expressed *Av*NifEN$^{Ec\text{-}L}$, a heterotetramer composed of α- and β-subunits of ~50 kDa and ~49 kDa, respectively. (**B**) Fe analyses of *Av*NifEN^{L} (black) and *Av*NifEN$^{Ec\text{-}L}$ (red). The L-cluster content of *Av*NifEN^{L} or *Av*NifEN$^{Ec\text{-}L}$ was calculated by subtracting 8 Fe atoms in the two permanent O-clusters (Fe_4S_4]) from the total amount of Fe atoms per NifEN tetramer. The relative L-cluster content of *Av*NifEN$^{Ec\text{-}L}$ (expressed in percentage), as compared to that of *Av*NifEN^{L} (set as 100%), is indicated in red font. (**C**) Activity analyses of *Av*NifEN^{L} (black) and *Av*NifEN$^{Ec\text{-}L}$ (red) in catalysis (*Catal.*) and M-cluster assembly (*Assem.*). The relative C_2H_2-reduction activities of *Av*NifEN$^{Ec\text{-}L}$ (expressed in percentage), as compared to those of *Av*NifEN^{L} (set as 100%), are indicated in red font. See table S1 for details on the Fe contents and specific activities of *Av*NifEN$^{Ec\text{-}L}$ and *Av*NifEN^{L}. (**D**) SDS-PAGE of the heterologously expressed *Av*NifH^{Ec}, a homodimer composed of two ~30 kDa subunits. (**E**) Fe analyses of *Av*NifH (black) and *Av*NifH^{Ec} (red). The relative Fe content of *Av*NifH^{Ec} (expressed in percentage), as compared to that of *Av*NifH (set as 100%), is indicated in red font. (**F**) Activity analyses of *Av*NifH (black) and *Av*NifH^{Ec} (red) in C_2H_2 reduction. The relative activity of *Av*NifH^{Ec} (expressed in percentage), as compared to that of *Av*NifH (set as 100%), is indicated in red font. See table S2 for details on the Fe contents and specific activities of *Av*NifH^{Ec} and *Av*NifH. Data in B, C, E and F are expressed as mean ± s.d. (*N*=3).

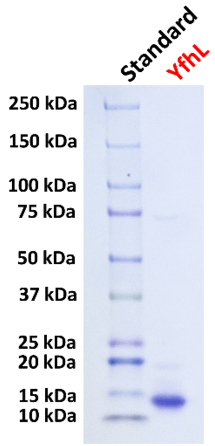

fig. S3. SDS-PAGE of YfhL. Shown is the YfhL protein purified from *E. coli* strain YM646EE with a molecular weight of ~11 kDa.

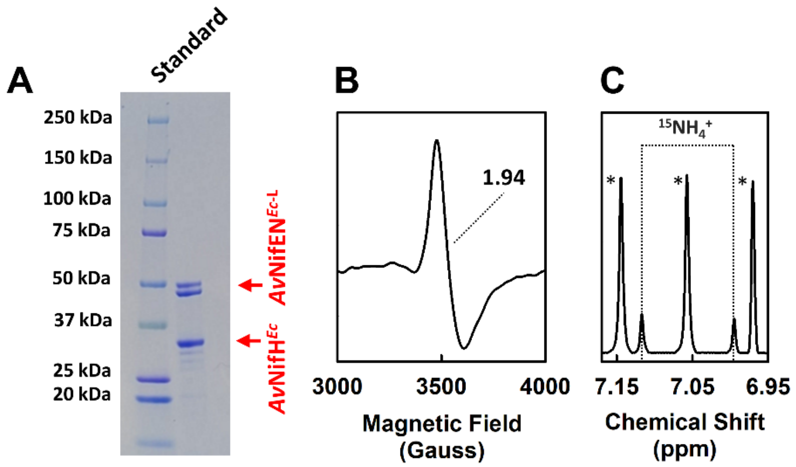

fig. S4. Biochemical and spectroscopic characterization of *Av*NifH^{Ec} and *Av*NifEN$^{Ec\text{-}L}$ expressed in the absence of YfhL. (**A**) SDS-PAGE analysis of *Av*NifH^{Ec} and *Av*NifEN$^{Ec\text{-}L}$ co-purified from YM634EE, a *yfhL*-deletion *E. coli* strain. (**B**) Perpendicular-mode EPR analysis of the IDS-oxidized *Av*NifEN$^{Ec\text{-}L}$ protein isolated from a *yfhL*-deletion background. (**C**) Frequency-selective pulse ^{1}NMR analysis of $^{15}NH_4^+$ generated upon ATP-dependent reduction of $^{15}N_2$ by *Av*NifH^{Ec} and *Av*NifEN$^{Ec\text{-}L}$ co-purified from YM634EE at a molar ration of ~1:1. Note that the triplet signals in the NMR spectra (labeled with *) represent the $^{14}NH_4^+$ background generated upon protein degradation.

Supplementary Tables

table S1. Specific activities and Fe contents of *Av*NifEN^{L} and *Av*NifEN$^{Ec\text{-}L}$

Protein	Specific Activity			
	Catalysis		Assembly	
	C_2H_2-reduction (to C_2H_4)		C_2H_2-reduction (to C_2H_4)	
	nmol product/ mg protein/min	*%*	*nmol product/ mg protein/min*	*%*
*Av*NifEN^{L}	33.3 ± 1.2	100 ± 3.7	529.1 ± 65.3	100 ± 12.3
*Av*NifEN$^{Ec\text{-}L}$	13.1 ± 0.7	39.3 ± 2.0	212.4 ± 12.4	40.1 ± 2.4
	**Metal content**			
	**Total Fe content**		**L-cluster content***	
	mol Fe/ mol protein	*%*	*mol Fe/ mol protein*	*%*
*Av*NifEN^{L}	18.5 ± 2.7	100 ± 14.6	10.5	100
*Av*NifEN$^{Ec\text{-}L}$	12.5 ± 1.7	67.6 ± 9.2	4.5	42.9

*Calculated by subtracting 8 mol Fe/mol protein, which represents the Fe content of the two permanent [Fe_4S_4] clusters (*i.e.*, the O-cluster) per NifEN tetramer, from the total Fe content.

table S2. Specific activities and Fe contents of *Av*NifH and *Av*NifH^{Ec}

Protein	Specific Activity	
	C_2H_2-reduction (to C_2H_4)	
	nmol product/ mg protein/min	*%*
*Av*NifH	2052 ± 118	100 ± 6
*Av*NifH^{Ec}	1621 ± 134	79 ± 7
	**Fe content**	
	mol Fe/ mol protein	*%*
*Av*NifH	3.9 ± 0.2	100 ± 4
*Av*NifH^{Ec}	3.1 ± 0.4	78 ± 9
